# Supplementary material for: Relative age effect? No “flipping” way! Apparatus dependent inverse relative age effects in elite, women’s artistic gymnastics
Source: PLoS One. 2021 Jun 28;16(6):e0253656. doi: 10.1371/journal.pone.0253656 (PMC8238206; doi:10.1371/journal.pone.0253656)
Supplement: S1 Table — (DOCX) [file pone.0253656.s001.docx]

**S1 Table. Summary of Country Representation within the Analysis**

| Country | Number of Gymnasts | | | | | | |
| --- | --- | --- | --- | --- | --- | --- | --- |
|  | International Elite Gymnasts | | | Apparatus Specialists | | | |
|  | Full Sample | Seniors | Juniors | Vault | Uneven Bars | Beam | Floor |
| Algeria | 4 | 2 | 1 | 0 | 0 | 0 | 0 |
| Argentina | 9 | 6 | 1 | 0 | 0 | 0 | 0 |
| Armenia | 1 | 0 | 0 | 0 | 0 | 0 | 0 |
| Australia | 18 | 12 | 1 | 0 | 2 | 1 | 2 |
| Austria | 12 | 8 | 0 | 0 | 0 | 2 | 0 |
| Azerbaijan | 6 | 3 | 0 | 1 | 0 | 0 | 1 |
| Bahamas | 2 | 0 | 0 | 0 | 0 | 0 | 0 |
| Belarus | 10 | 4 | 3 | 1 | 1 | 0 | 0 |
| Belgium | 21 | 8 | 6 | 0 | 2 | 4 | 4 |
| Bolivia | 3 | 2 | 0 | 0 | 0 | 0 | 0 |
| Brazil | 12 | 9 | 1 | 2 | 0 | 2 | 4 |
| Bulgaria | 7 | 6 | 0 | 0 | 0 | 0 | 0 |
| Canada | 32 | 15 | 2 | 3 | 0 | 4 | 2 |
| Cayman Islands | 2 | 1 | 0 | 0 | 0 | 0 | 0 |
| Chile | 3 | 3 | 0 | 0 | 0 | 0 | 0 |
| China | 27 | 19 | 6 | 4 | 9 | 14 | 7 |
| Chinese Taipei | 8 | 8 | 0 | 0 | 0 | 0 | 0 |
| Colombia | 9 | 8 | 0 | 0 | 0 | 0 | 1 |
| Costa Rica | 5 | 4 | 1 | 0 | 0 | 0 | 0 |
| Croatia | 5 | 3 | 0 | 1 | 0 | 0 | 0 |
| Cuba | 3 | 2 | 0 | 0 | 0 | 0 | 0 |
| Cyprus | 3 | 2 | 0 | 0 | 0 | 0 | 0 |
| Czech Republic | 12 | 8 | 0 | 1 | 1 | 0 | 0 |
| Denmark | 10 | 5 | 1 | 0 | 0 | 0 | 0 |
| Dominican Republic | 1 | 1 | 0 | 1 | 0 | 0 | 0 |
| Ecuador | 1 | 1 | 0 | 0 | 0 | 0 | 0 |
| Egypt | 7 | 5 | 1 | 0 | 0 | 0 | 0 |
| Finland | 10 | 7 | 2 | 0 | 0 | 0 | 0 |
| France | 25 | 11 | 4 | 4 | 7 | 8 | 6 |
| Georgia | 3 | 2 | 0 | 0 | 0 | 0 | 0 |
| Germany | 22 | 17 | 2 | 5 | 6 | 3 | 3 |
| Great Britain | 37 | 16 | 5 | 6 | 7 | 6 | 9 |
| Greece | 8 | 5 | 1 | 0 | 0 | 2 | 0 |
| Guatemala | 3 | 2 | 1 | 0 | 0 | 0 | 0 |
| Honduras | 1 | 0 | 0 | 0 | 0 | 0 | 0 |
| Hong Kong | 3 | 3 | 0 | 0 | 0 | 0 | 0 |
| Hungary | 15 | 7 | 1 | 7 | 1 | 1 | 1 |
| Iceland | 12 | 8 | 2 | 0 | 0 | 0 | 0 |
| India | 3 | 3 | 0 | 1 | 0 | 0 | 0 |
| Indonesia | 3 | 3 | 0 | 0 | 0 | 0 | 0 |
| Ireland | 7 | 3 | 2 | 0 | 0 | 0 | 0 |
| Israel | 5 | 5 | 0 | 2 | 0 | 1 | 0 |
| Italy | 34 | 20 | 2 | 6 | 6 | 5 | 6 |
| Jamaica | 1 | 1 | 0 | 0 | 0 | 0 | 0 |
| Japan | 21 | 19 | 0 | 1 | 3 | 3 | 2 |
| Kazakhstan | 5 | 5 | 0 | 0 | 0 | 0 | 0 |
| Latvia | 8 | 6 | 1 | 0 | 0 | 0 | 0 |
| Lithuania | 3 | 2 | 1 | 0 | 0 | 0 | 0 |
| Luxembourg | 2 | 0 | 0 | 0 | 0 | 0 | 0 |
| Malaysia | 5 | 4 | 0 | 0 | 0 | 0 | 0 |
| Malta | 2 | 1 | 0 | 0 | 0 | 0 | 0 |
| Mexico | 14 | 9 | 2 | 2 | 0 | 0 | 0 |
| Monaco | 1 | 0 | 0 | 0 | 0 | 0 | 0 |
| Mongolia | 1 | 0 | 0 | 0 | 0 | 0 | 0 |
| Morocco | 2 | 0 | 0 | 0 | 0 | 0 | 0 |
| Namibia | 3 | 0 | 0 | 0 | 0 | 0 | 0 |
| Netherlands | 19 | 13 | 3 | 4 | 4 | 3 | 4 |
| New Zealand | 11 | 7 | 0 | 0 | 0 | 0 | 0 |
| North Korea | 11 | 6 | 0 | 3 | 0 | 1 | 0 |
| Norway | 9 | 8 | 1 | 0 | 0 | 0 | 0 |
| Panama | 2 | 0 | 0 | 0 | 0 | 0 | 0 |
| Peru | 3 | 2 | 0 | 0 | 0 | 0 | 0 |
| Philippines | 2 | 1 | 0 | 0 | 0 | 0 | 0 |
| Poland | 8 | 5 | 0 | 1 | 0 | 2 | 1 |
| Portugal | 7 | 6 | 0 | 0 | 0 | 0 | 0 |
| Puerto Rico | 4 | 2 | 1 | 0 | 0 | 0 | 0 |
| Qatar | 1 | 1 | 0 | 0 | 0 | 0 | 0 |
| Romania | 25 | 11 | 5 | 5 | 3 | 12 | 11 |
| Russia | 34 | 23 | 7 | 14 | 15 | 17 | 14 |
| Serbia | 4 | 4 | 0 | 0 | 0 | 0 | 0 |
| Singapore | 8 | 5 | 1 | 0 | 0 | 0 | 0 |
| Slovakia | 8 | 7 | 1 | 0 | 0 | 0 | 0 |
| Slovenia | 8 | 7 | 1 | 1 | 0 | 0 | 0 |
| South Africa | 9 | 7 | 1 | 0 | 0 | 0 | 0 |
| South Korea | 13 | 11 | 1 | 2 | 0 | 0 | 0 |
| Spain | 15 | 10 | 3 | 0 | 2 | 1 | 4 |
| Sri Lanka | 13 | 0 | 1 | 0 | 0 | 0 | 0 |
| Sweden | 9 | 4 | 1 | 0 | 2 | 0 | 1 |
| Switzerland | 14 | 11 | 0 | 2 | 0 | 4 | 1 |
| Syria | 1 | 1 | 0 | 0 | 0 | 0 | 0 |
| Trinidad and Tobago | 2 | 0 | 0 | 0 | 0 | 0 | 0 |
| Turkey | 7 | 6 | 1 | 0 | 0 | 0 | 0 |
| Ukraine | 9 | 7 | 1 | 2 | 8 | 9 | 5 |
| United States | 56 | 15 | 9 | 6 | 12 | 12 | 15 |
| Uruguay | 1 | 0 | 0 | 0 | 0 | 0 | 0 |
| Uzbekistan | 5 | 2 | 1 | 1 | 0 | 0 | 1 |
| Venezuela | 4 | 2 | 0 | 0 | 10 | 0 | 0 |
| Vietnam | 6 | 4 | 1 | 1 | 0 | 0 | 0 |
